# Supplementary material for: Multi‐dataset identification of innovative feature genes and molecular mechanisms in keratoconus
Source: J Cell Mol Med. 2024 Sep 19;28(18):e70079. doi: 10.1111/jcmm.70079 (PMC11412914; doi:10.1111/jcmm.70079)
Supplement: Supplementary file 2 — Material S2. [file JCMM-28-e70079-s002.zip › 7.GSEA analysis/6.SNORA74B/gsea_report_for_h_1678852949716.html]

Report for h 1678852949716 [GSEA]

| GS  follow link to MSigDB | GS DETAILS | SIZE | ES | NES | NOM p-val | FDR q-val | FWER p-val | RANK AT MAX | LEADING EDGE || 1 | KEGG\_RIBOSOME | Details ... | 85 | 0.29 | 1.07 | 0.331 | 1.000 | 0.996 | 23307 | tags=99%, list=67%, signal=300% |
| 2 | KEGG\_PORPHYRIN\_AND\_CHLOROPHYLL\_METABOLISM | Details ... | 32 | 0.33 | 1.06 | 0.409 | 1.000 | 0.998 | 7776 | tags=25%, list=22%, signal=32% |
| 3 | KEGG\_PHENYLALANINE\_METABOLISM | Details ... | 17 | 0.36 | 1.01 | 0.473 | 1.000 | 1.000 | 5832 | tags=29%, list=17%, signal=35% |
| 4 | KEGG\_RNA\_DEGRADATION | Details ... | 51 | 0.18 | 0.97 | 0.573 | 1.000 | 1.000 | 28396 | tags=100%, list=82%, signal=548% |
| 5 | KEGG\_PENTOSE\_AND\_GLUCURONATE\_INTERCONVERSIONS | Details ... | 20 | 0.36 | 0.96 | 0.497 | 1.000 | 1.000 | 10337 | tags=40%, list=30%, signal=57% |
| 6 | KEGG\_GLYCINE\_SERINE\_AND\_THREONINE\_METABOLISM | Details ... | 31 | 0.27 | 0.91 | 0.665 | 1.000 | 1.000 | 3647 | tags=19%, list=11%, signal=22% |
| 7 | KEGG\_PROTEIN\_EXPORT | Details ... | 22 | 0.28 | 0.91 | 0.648 | 1.000 | 1.000 | 25096 | tags=100%, list=72%, signal=360% |
| 8 | KEGG\_TASTE\_TRANSDUCTION | Details ... | 52 | 0.31 | 0.90 | 0.620 | 1.000 | 1.000 | 4608 | tags=29%, list=13%, signal=33% |
| 9 | KEGG\_GLYOXYLATE\_AND\_DICARBOXYLATE\_METABOLISM | Details ... | 16 | 0.31 | 0.90 | 0.570 | 1.000 | 1.000 | 3587 | tags=13%, list=10%, signal=14% |
| 10 | KEGG\_HOMOLOGOUS\_RECOMBINATION | Details ... | 26 | 0.27 | 0.87 | 0.590 | 1.000 | 1.000 | 1591 | tags=8%, list=5%, signal=8% |
| 11 | KEGG\_TERPENOID\_BACKBONE\_BIOSYNTHESIS | Details ... | 15 | 0.32 | 0.87 | 0.630 | 1.000 | 1.000 | 13502 | tags=60%, list=39%, signal=98% |
| 12 | KEGG\_NEUROACTIVE\_LIGAND\_RECEPTOR\_INTERACTION | Details ... | 270 | 0.22 | 0.79 | 0.998 | 1.000 | 1.000 | 5215 | tags=19%, list=15%, signal=22% |
| 13 | KEGG\_GLYCOSAMINOGLYCAN\_DEGRADATION | Details ... | 21 | 0.25 | 0.79 | 0.730 | 1.000 | 1.000 | 1272 | tags=5%, list=4%, signal=5% |
| 14 | KEGG\_ASCORBATE\_AND\_ALDARATE\_METABOLISM | Details ... | 17 | 0.28 | 0.76 | 0.830 | 1.000 | 1.000 | 3661 | tags=18%, list=11%, signal=20% |
| 15 | KEGG\_GLYCOSYLPHOSPHATIDYLINOSITOL\_GPI\_ANCHOR\_BIOSYNTHESIS | Details ... | 25 | 0.24 | 0.75 | 0.705 | 1.000 | 1.000 | 26316 | tags=100%, list=76%, signal=413% |
| 16 | KEGG\_OOCYTE\_MEIOSIS | Details ... | 111 | 0.14 | 0.67 | 0.925 | 1.000 | 1.000 | 7434 | tags=14%, list=21%, signal=18% |
| 17 | KEGG\_SPLICEOSOME | Details ... | 125 | 0.15 | 0.64 | 0.931 | 1.000 | 1.000 | 24868 | tags=97%, list=72%, signal=340% |
| 18 | KEGG\_PEROXISOME | Details ... | 78 | 0.14 | 0.57 | 0.953 | 1.000 | 1.000 | 3063 | tags=6%, list=9%, signal=7% |
| 19 | KEGG\_GLYCOSPHINGOLIPID\_BIOSYNTHESIS\_GANGLIO\_SERIES | Details ... | 15 | 0.19 | 0.53 | 0.969 | 1.000 | 1.000 | 13934 | tags=53%, list=40%, signal=89% |
| 20 | KEGG\_PRIMARY\_IMMUNODEFICIENCY | Details ... | 35 | 0.19 | 0.51 | 1.000 | 1.000 | 1.000 | 5304 | tags=11%, list=15%, signal=13% |
| 21 | KEGG\_PROTEASOME | Details ... | 43 | 0.12 | 0.41 | 1.000 | 0.999 | 1.000 | 22841 | tags=95%, list=66%, signal=278% |
Table: Gene sets enriched in phenotype **h (8 samples) **[plain text format]****
